# CaMKII, that binds with ligustilide, as a potential drug target of Suxiao jiuxin pill, a traditional Chinese medicine to dilate thoracic aorta

**DOI:** 10.1002/ctm2.907

**Published:** 2022-06-09

**Authors:** Yujie Lu, Jie Ji, Simeng Chu, Fukui Shen, Wen Yang, Wei Lei, Min Jiang, Gang Bai

**Affiliations:** ^1^ State Key Laboratory of Medicinal Chemical Biology, College of Pharmacy and Tianjin Key Laboratory of Molecular Drug Research Nankai University Tianjin China; ^2^ First Teaching Hospital of Tianjin University of Traditional Chinese Medicine Tianjin China; ^3^ National Clinical Research Center for Chinese Medicine Acupuncture and Moxibustion Tianjin China; ^4^ Tianjin University of Traditional Chinese Medicine Tianjin China

Dear Editor,

Vasodilation is one of the main means to treat cardiovascular diseases, such as heart failure, cardiogenic shock and severe hypertension.^1^ Although blocking voltage‐gated calcium channels with specific antagonists stands as an optimal therapeutic strategy,^2^ targeting Ca^2+^/calmodulin‐dependent protein kinase II (CaMKII) may also be a suitable approach, as this enzyme also plays an important role in the regulation of Ca^2+^‐related physiological functions^3^, ^4^ and cardiovascular diseases.^5^, ^6^ Herein, we reveal a novel mechanism of CaMKII covalent inhibition by one kind of the phthalides present in Suxiao Jiuxin pills (SX), a traditional Chinese medicine frequently used for the treatment of cardiovascular disease.

A head‐to‐head randomized clinical comparison of SX and atorvastatin calcium (At‐Ca) in patients with atherosclerosis showed that SX significantly increased cardiac output, while At‐Ca mainly lowered lipid overload, indicating that these compounds ameliorate clinical symptoms through different mechanisms (Figure 1A–H, Tables S1 andS2). SX treatment does not affect heart rate and has little effect on myocardial contractility, so it may increase cardiac output by dilating the thoracic aorta (Figures S1, S2). To identify the potential vasodilator targets of SX, a cellular thermal shift assay (CETSA) coupled with iTRAQ‐based differential proteomic analysis was performed. The abundance of 199 unique proteins (among which 142 were upregulated and 57 were downregulated) was significantly altered after exposure to SX (Figure 1I). Among them, only one potential target protein was identified, namely, CaMKIIδ which participates in cardiovascular contraction by regulating the calcium signalling pathway (Figure 1J–L). Additionally, CETSA results demonstrated that the SX extract effectively improved the thermal stability of CaMKII in human vascular smooth muscle cells (VSMCs; Figure 1 M).

**FIGURE 1:**
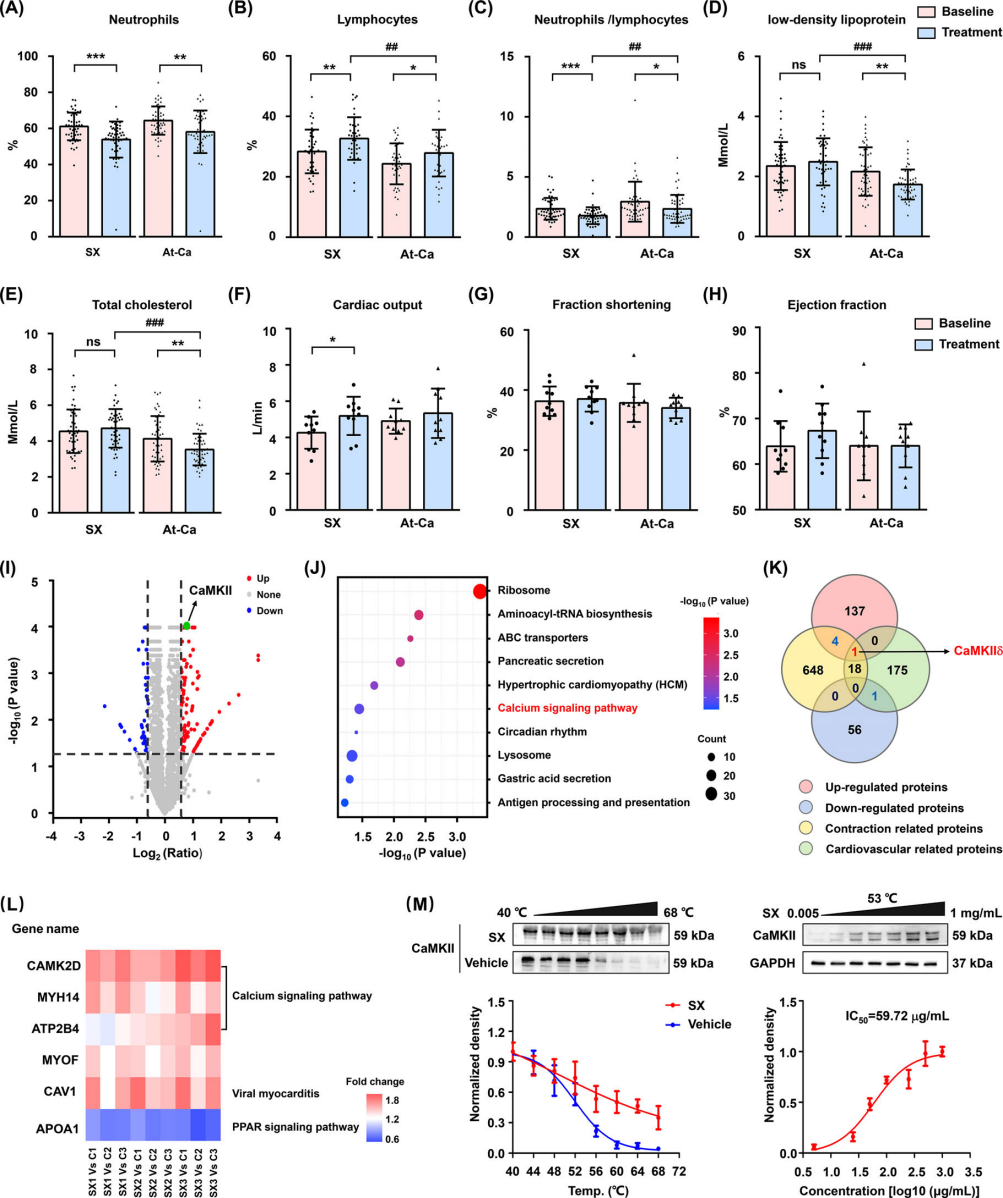
The clinical efficacy evaluation of Suxiao Jiuxin pills (SX) and differential proteomics assay for identifying the potential target in vasodilator activity. (A–E) Patients treated with SX (40 mg a pill, 6 pills a time, three times a day, sublingually) or atorvastatin calcium tablets (At‐Ca, 10 mg tablet a time, once a day, orally) for 2 weeks underwent routine blood examination, including neutrophils (NEUT), lymphocytes (LYMPH), and the ratio of neutrophils to lymphocytes (NEUT/LYMPH), and blood lipid examination including low‐density lipoprotein (LDL) and total cholesterol (TC) contents, before and after treatment (*n* = 50). (F–H) The cardiac output (CO), fraction shortening (FS), and ejection fraction (EF) of patients were measured using colour Doppler echocardiography (*n* = 10). (I) Volcanic map of the quantitative proteins identification by cellular thermal shift assay (CETSA) combined with iTRAQ‐based proteomic analysis in human vascular smooth muscle cell (VSMCs) lysates treated with SX extract (1 mg/ml). (J) Top 10 significantly enriched pathways using the Kyoto Encyclopaedia of Genes and Genomes (KEGG) database. (K) Venn diagram analysis of SX targets in relaxing cardiovascular activity. The red intersection is the common target CaMKIIδ (gene name: CAMK2D). (L) The heat map of cluster analysis illustrates the iTRAQ results of six contraction or cardiovascular‐related proteins. (M) SX extract (1 mg/ml) treatment increased the thermal stability of CaMKII in VSMCs as measured by temperature (left panel) or dose (right panel) dependent CETSA (*n *= 3). ^*^ *p *< .05 and ^**^ *p *< .01 compared with baseline; ^##^ *p *< .01 and ^###^ *p *< .001 compared between two groups by a two‐sided Student's *t*‐test

Subsequently, ultra‐performance liquid chromatography coupled with CaMKII‐mediated calcium antagonist screening was conducted to identify the active pharmaceutical ingredient in SX (Figure 2A). The results revealed that the two major components of SX, ligustilide (Lig) and senkyunolide A (Sen A), exhibited Ca^2+^‐inhibitory activity by acting on CaMKII (Figures 2B, S3). Because Lig was more abundant in SX than Sen A (Figure S4, Table S3), an alkynyl‐modified Lig probe and its fluorophore tracer were synthesized and used to verify the interaction between Lig and CaMKII (Figures 2C, S5–S9). VSMCs staining showed that the fluorescence of the Lig‐modified tracer partly overlapped with that of the CaMKII antibody, while the overlapping position could be replaced by 10‐fold free Lig and Sen A competition (Figures 2D, S10). Moreover, an in‐gel imaging assay of 293T cells overexpressing CaMKII and VSMCs indicated that phthalides with double bonds at the C6/C7 might be the key pharmacophores involved in the covalent binding to CaMKII (Figure 2E,F). Furthermore, CETSA results indicated that Lig and Sen A had a similar effect on CaMKII, whereas senkyunolide I (Sen I) had no significant effect (Figure 2G).

**FIGURE 2:**
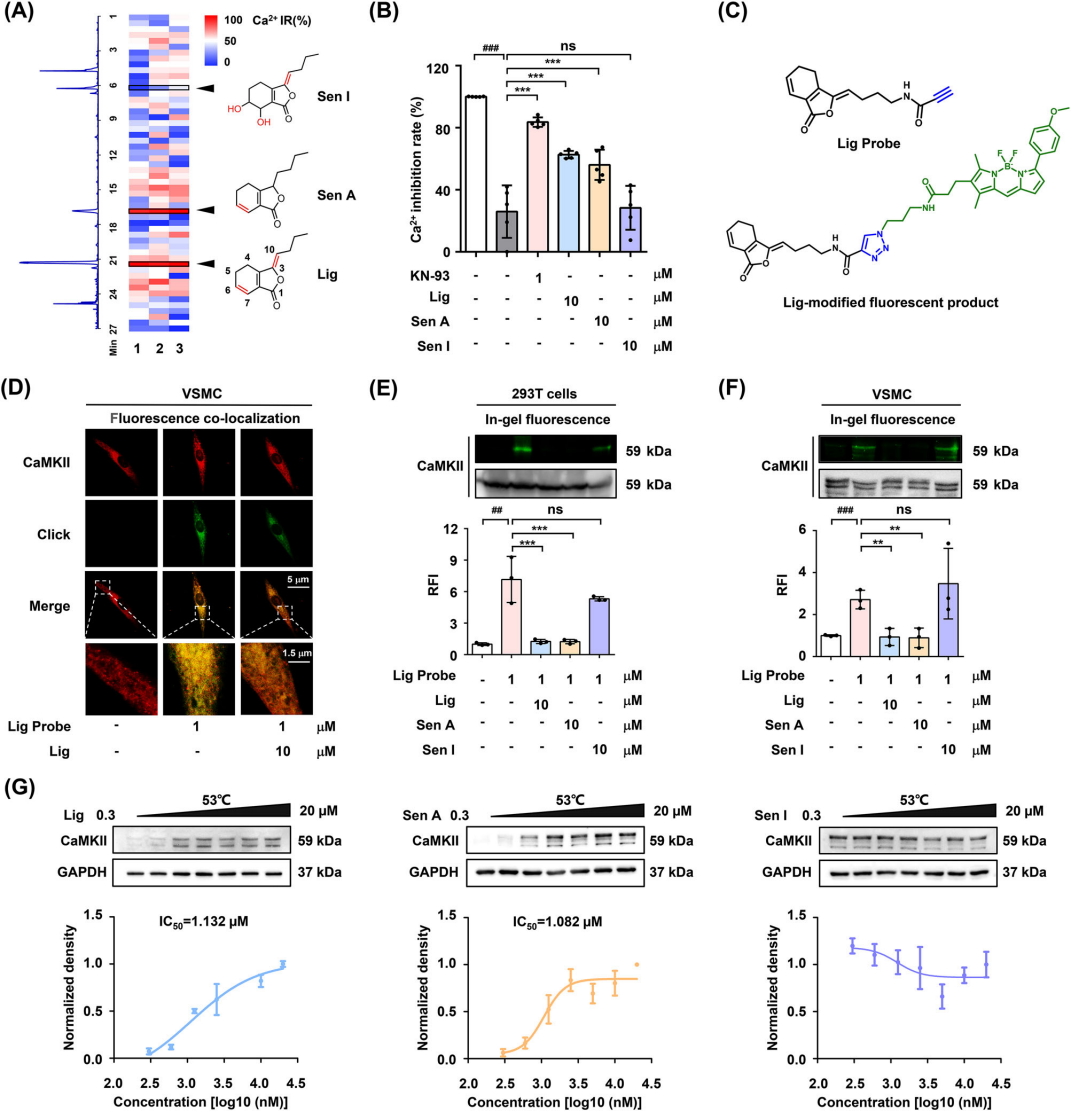
Phthalides with C6/C7 double bonds in Suxiao Jiuxin pills (SX) are the key compounds for CaMKII inhibition. (A) UPLC combined with dual‐luciferase reporter assays was used to screen the CaMKII‐dependent calcium antagonists from SX extract (*n *= 3). The 293T cells were co‐transfected with CaMKIIG plasmid, Ca^2+^ luciferase reporter plasmid PGL 4.30, and Renilla luciferase plasmid (50:25:1, m/m/m) for 22 h. The cells were stimulated with ionomycin (1 mM) and phorbol 12‐myristate 13‐acetate (1 mg/mL) for 6 h, and simultaneously treated with drugs. (B) Confirmatory test of active pharmaceutical ingredient (API) for intracellular calcium inhibition activity, and CaMKII competitive inhibitor KN‐93 as a positive control (*n *= 5). (C) Chemical structure of Lig probe and its fluorescent tracer, which conjugated with boron‐dipyrromethene tetramethylrhodamine (BDP TMR) azide via click reaction. (D) Co‐localization of CaMKII (pseudo red) and Lig probe (pseudo green) in VSMCs. The distribution of merged yellow in the Lig probe group could be partial replaced by 10‐fold free Lig competition, the Pearson coefficient (PC) value dropped from ∼0.91 to ∼ 0.32. (E and F) In‐gel imaging analysis for CaMKII protein labelled with Lig probe and BDP TMR tracer (upper panel) in the 293T cells with over‐expression of CaMKII or VSMCs. Western blot was used as a control of CaMKII protein (lower panel). The cells were all treated with Lig probe (1 μM) in the absence or presence of 10 μM competitive agents, including Lig, Sen A or Sen I. (G) The thermal stability of CaMKII at 53°C in VSMCs treated with different doses of Lig, Sen A and Sen I (0.3, 0.6, 1.25, 2.5, 5, 10 and 20 μM) was measured with dose‐dependent CETSA (*n* = 3). Bars represent the mean ± SD. ^##^ *p *< .01 and ^###^ *p *< .001 compared with the control group by a two‐sided Student's *t*‐test. ^**^ *p *< .01 and ^***^ *p *< .001 compared with the model group by one‐way analysis of variance (ANOVA)

In humans, four subtypes of CaMKII share the N‐terminal catalytic/autoregulatory domain and a C‐terminal association domain, which is necessary for the assembly of the CaMKII holoenzyme complex (Figure 3A).^7^, ^8^ To identify the binding site of Lig in CaMKII, the recombinant wild‐type human CaMKIIγ protein (residues 1–271) was expressed and purified (Figure S11), then simultaneously incubated with Lig probe and liver tissue lysates. The results of the in‐gel imaging assay revealed that only Lig and Sen A covalently bound to CaMKII after liver metabolism (Figures 3B, S12, S13). It is well‐established that the C6/C7 double bond of Lig is metabolized into an epoxy group in vivo.^9^, ^10^ Protein mass spectrometry identification revealed that the epoxidised metabolite 7‐epoxyligustilide (E‐Lig) attacks the thiol group of Cys116 on the CaMKII catalytic domain (Figure 3C). Molecular docking indicated that the epoxy group of E‐Lig was destroyed, followed by a nucleophilic addition interaction at Cys116 of CaMKIIγ. Meanwhile, the newly formed hydroxyl group established stable hydrogen bond interactions with the Asp‐112 and His‐115 residues (Figure 3D).

**FIGURE 3:**
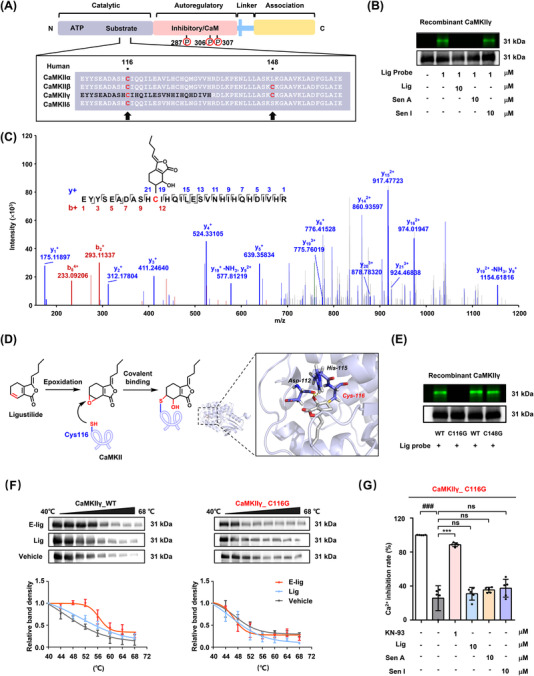
E‐Lig covalently binds to CaMKII at Cys116 via nucleophilic addition. (A) Comparison of the main sequences of human members of the CaMKII family. (B) The C6/C7 double bond of Lig was proposed to be a major pharmacophore for Lig binding to CaMKII. Recombinant CaMKIIγ was co‐incubated with Lig probe in the absence or presence of Lig, Sen A and Sen I (10 μM) at 4°C for 12 h under the catalysis of liver tissue lysates, and the products were used for in‐gel imaging. (C) LC–MS/MS analysis of the recombinant CaMKIIγ protein incubated with E‐Lig for 12 h. E‐Lig binds to the Cys116 residue of CaMKIIγ. (D) The proposed covalent binding mode of Lig to CaMKII (left panel). And molecular modelling of E‐Lig covalently bound to the Cys116 of CaMKIIγ (PDB: 2V7O) (right panel). E‐Lig is displayed in sticks and coloured by atomic type, in which the carbon atom is gray. The group that reacted with Cys116 of CaMKIIγ is shown in yellow. (E) In‐gel imaging of wild‐type CaMKIIγ and the two mutants (C116G and C148G). The proteins were incubated with the Lig probe (1 μM) catalysed by liver tissue lysates at 4°C for 12 h. (F) E‐Lig metabolite increased the thermal stability of wild‐type CaMKIIγ but had no effect on the C116G mutant, as measured by thermal shift assay (*n* = 3). (G) The calcium antagonistic activity of the compound was determined in 293T cells with high expression of CaMKIIγ_C116G protein. Bars represent the mean ± SD. ^###^ *p *< .001 compared with the control group by a two‐sided Student's *t*‐test. ^***^ *p *< .001 compared with the model group by one‐way analysis of variance (ANOVA)

Moreover, CaMKIIγ protein with mutated cysteine residues (C116G and C148G) was utilized to test this hypothesis by in‐gel imaging assays. As predicted, E‐Lig selectively covalently bound to Cys116 independently of Cys148 (Figure 3E). Thermal stability analysis also supported that only the metabolite of Lig (E‐Lig) could covalently bind to CaMKII at Cys116, suggesting this residue may be a druggable site (Figures 3F, S14). Once the Cys116 residue was destroyed, the calcium antagonistic activity of Lig and Sen A in 293T cells overexpressing C116G protein also disappeared (Figures 3G, S15).

To verify the vasodilator effect of Lig in vivo, transthoracic echocardiography was performed to detect the degree of systolic dysfunction in atherosclerotic ApoE^−/−^ mice. Lig treatment effectively reversed the decrease in cardiac output and increased fractional shortening and ejection fraction in a dose‐dependent manner (Figures 4A, S16). Co‐localization imaging of living tissues revealed that the oral Lig probe mainly targeted CaMKII in the VSMCs of the thoracic aorta (Figure 4B). After oral administration of Lig, the relative fluorescence intensity of phosphorylated CaMKII in the thoracic aorta decreased significantly (Figure 4C). In addition, an experiment with KCl‐induced isolated rat thoracic aortic ring indicated that Lig exerts a dramatic vasodilatory effect by inhibiting CaMKII autophosphorylation and its downstream myosin light chain phosphorylation (Figures 4D,E, S17).

**FIGURE 4:**
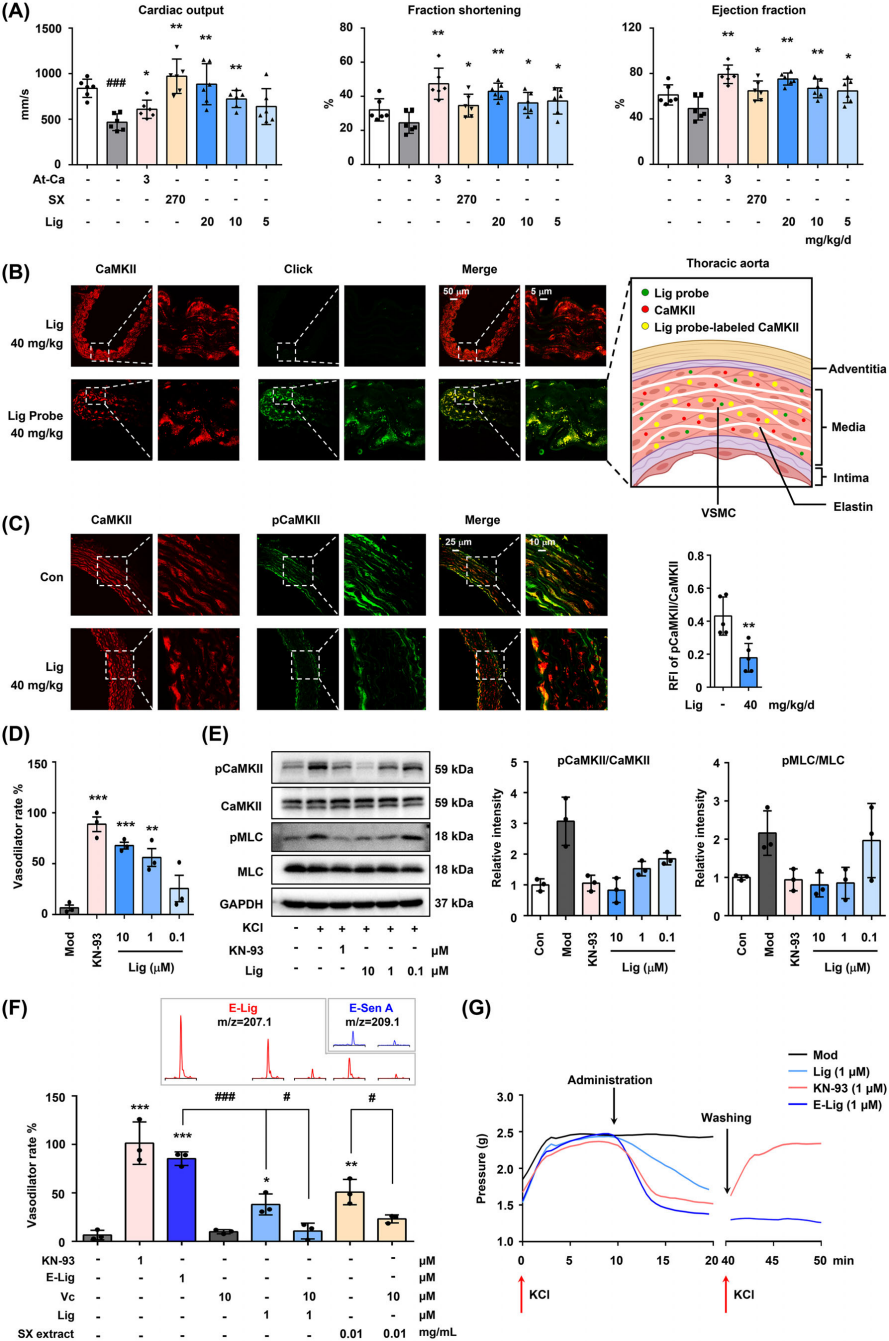
Lig alleviates vascular and cardiac dysfunction by specifically inhibiting the phosphorylation of CaMKII in VSMCs of thoracic aorta after metabolism to epoxide. (A) The cardiac output (CO), fraction shortening (FS) and ejection fraction (EF) were measured in wild‐type control C57BL/6J mice treated with normal saline and ApoE^−/−^ mice treated with normal saline, At‐Ca, Suxiao Jiuxin pills (SX) and three doses of Lig (20, 10, 5 mg/kg) for 12 weeks, respectively. (B) The co‐localization imaging of CaMKII (pseudo red) and Lig probe (pseudo green) in thoracic aorta sections of mice, which were treated with Lig probe (40 mg/kg) for 7 days (left panel). The schematic diagram of the thoracic aorta, where Lig binds primarily to CaMKII of the VSMCs in the media (right panel). (C) Immunohistochemical staining for the detection of p‐CaMKII (pseudo green) and CaMKII (pseudo red) in the thoracic aorta sections of mice, which were treated with Lig (40 mg/kg) for 7 days (*n* = 5). (D) Lig regulated the vasodilation of isolated rat thoracic aorta in a vasodilatory effect test. (E) Lig attenuated KCl‐induced phosphorylation of CaMKII and MLC in isolated rat thoracic aorta. (F) The vasodilator effects of KN‐93, E‐Lig, Lig and SX API extract in isolated thoracic aorta of rats (lower panel). Extracted ion chromatograms of E‐Lig and epoxidised Sen A (E‐Sen A) in the incubation solution of thoracic aorta (upper panel), which correspond to the column chart directly below in each group. (G) Time‐dependent monitoring of vascular relaxation regulated by E‐Lig and KN‐93 in an isolated thoracic aorta of rats. At 40 min, washed off the incubated drug, re‐stimulated with KCl, and continued to record the contraction of the thoracic aortas. Bars represent the mean ± SD (*n* = 3). Significant differences between two groups were assessed using *t*‐tests, and analysis of multiple groups was performed using one‐way analysis of variance (ANOVA). ^*^ *p *< .05, ^**^ *p *< .01 and ^***^ *p *< .001 compared with the model group

Next, the benefit of the covalent bound between E‐Lig and CaMKII on the vasodilator activity was evaluated using an isolated aortic ring test. The vasodilator effect of E‐Lig was significantly stronger than that of Lig at the same dose. Remarkably, the effect could be suppressed by a reducing environment (Figure 4F), because the addition of vitamin C (Vc) inhibited the generation of the epoxidised metabolites, both in the Lig and SX extracts. Compared with the CaMKII competitive inhibitor KN‐93, the irreversible binding of E‐Lig was more enduring for vasodilator activity (Figure 4G).

In conclusion, as a key active component of SX, the epoxidised metabolite of Lig covalently binds to the Cys116 of CaMKII in VSMCs of the thoracic aorta, inhibits the autophosphorylation of CaMKII, and exerts a long‐lasting vasodilator effect. Our findings reveal a novel mechanism of inhibiting CaMKII phosphorylation via a nucleophilic addition reaction, providing insights into the development of a potentially promising drug for the treatment of cardiovascular diseases.

## CONFLICT OF INTEREST

The authors declare that there is no conflict of interest that could be perceived as prejudicing the impartiality of the research reported.

## Supporting information


**Table S1** Baseline characteristics of 100 patients included in the analyses
**Figure S1** Enrollment of participants and study flow
**Table S2** Baseline characteristics of 10 patients in each group
**Figure S2** Patients treated with SX (6 pills a time, 40 mg a pill, three times a day, sublingually) or atorvastatin calcium tablets (At‐Ca, 10 mg tablet a time, once a day, orally) for 2 weeks underwent high‐density lipoprotein (HDL) test (*n* = 50), and heart rate (HR) and representative M‐mode images of patients were measured using colour Doppler echocardiography (*n* = 10), before and after treatment
**Figure S3** siRNA interference assay combined with live‐cell imaging analysis reveal that the activity of Lig is mediated by CaMKII
**Figure S4** Lig and SenA in SX extract was analysed by UPLC
**Table S3** The bioactive components in SX extract were quantitatively analysed
**Figure S5** The detailed synthetic route of Lig‐PAL
**Figure S6** ^1^H NMR spectra for Lig‐PAL
**Figure S7** The detailed synthetic route of Lig probe
**Figure S8** The high‐resolution mass spectrometry (HRMS) of Lig probe
**Figure S9** The effects of Lig and Lig probe on CaMKII‐mediated Ca^2+^ antagonism
**Figure S10** Co‐localization of CaMKII (pseudo red) and Lig probe (pseudo green) in VSMCs, and Pearson coefficient (PC) analysis of the merged images
**Figure S11** The expression and purification of CaMKIIγ protein (residues 1‐271). (A) SDS‐PAGE analysis of CaMKIIγ protein
**Figure S12** Lig binds to CaMKIIγ after metabolism in liver tissue and liver microsomes
**Figure S13** In‐gel imaging for irreversible binding assay of Lig to CaMKIIγ
**Figure S14** E‐Lig metabolite increases the thermal stability of the C148G mutant CaMKIIγ, as measured by thermal shift assay (*n *= 3)
**Figure R15** Transfection of WT and C116G CaMKII plasmid into HEK293 cells induces high‐expression of WT and C116G protein
**Figure S16** Experimental procedure and representative M‐mode images were captured in wild‐type control C57BL/6J mice treated with normal saline and ApoE^−/−^ mice treated with normal saline, At‐Ca, SX and three doses of Lig, respectively
**Figure S17** Time‐dependent monitoring of vascular relaxation regulated by Lig and KN‐93 in an isolated thoracic aorta of rats
