# Supplementary material for: CaMKII, that binds with ligustilide, as a potential drug target of Suxiao jiuxin pill, a traditional Chinese medicine to dilate thoracic aorta
Source: Clin Transl Med. 2022 Jun 9;12(6):e907. doi: 10.1002/ctm2.907 (PMC9178388; doi:10.1002/ctm2.907)
Supplement: Supplementary file 1 — Table S1 Baseline characteristics of 100 patients included in the analyses Figure S1 Enrollment of participants and study flow Table S2 Baseline characteristics of 10 patients in each group Figure S2 Patients treated with SX (6 pills a time, 40 mg a pill, three times a day, sublingually) or atorvastatin calcium tablets (At‐Ca, 10 mg tablet a time, once a day, orally) for 2 weeks underwent high‐density lipoprotein (HDL) test (n = 50), and heart rate (HR) and representative M‐mode images of patients were measured using colour Doppler echocardiography (n = 10), before and after treatment Figure S3 siRNA interference assay combined with live‐cell imaging analysis reveal that the activity of Lig is mediated by CaMKII Figure S4 Lig and SenA in SX extract was analysed by UPLC Table S3 The bioactive components in SX extract were quantitatively analysed Figure S5 The detailed synthetic route of Lig‐PAL Figure S6 1H NMR spectra for Lig‐PAL Figure S7 The detailed synthetic route of Lig probe Figure S8 The high‐resolution mass spectrometry (HRMS) of Lig probe Figure S9 The effects of Lig and Lig probe on CaMKII‐mediated Ca2+ antagonism Figure S10 Co‐localization of CaMKII (pseudo red) and Lig probe (pseudo green) in VSMCs, and Pearson coefficient (PC) analysis of the merged images Figure S11 The expression and purification of CaMKIIγ protein (residues 1‐271). (A) SDS‐PAGE analysis of CaMKIIγ protein Figure S12 Lig binds to CaMKIIγ after metabolism in liver tissue and liver microsomes Figure S13 In‐gel imaging for irreversible binding assay of Lig to CaMKIIγ Figure S14 E‐Lig metabolite increases the thermal stability of the C148G mutant CaMKIIγ, as measured by thermal shift assay (n = 3) Figure R15 Transfection of WT and C116G CaMKII plasmid into HEK293 cells induces high‐expression of WT and C116G protein Figure S16 Experimental procedure and representative M‐mode images were captured in wild‐type control C57BL/6J mice treated with normal saline and ApoE−/− mice treated with no [file CTM2-12-e907-s001.docx]

**Supporting Information**

**CaMKII, that binds with ligustilide, as a potential drug target of Suxiao jiuxin pill, a traditional Chinese medicine to dilate thoracic aorta**

Yujie Lu^1,^ **^†^**, Jie Ji^2, 3,^ **^†^**, Simeng Chu^1^, Fukui Shen^1^, Wen Yang^1^, Wei Lei^4^, Min Jiang^1,^ *, Gang Bai^1,^ *

*^1^ State Key Laboratory of Medicinal Chemical Biology, College of Pharmacy and Tianjin Key Laboratory of Molecular Drug Research, Nankai University, Tianjin 300353, China*

*^2^ First Teaching Hospital of Tianjin University of Traditional Chinese Medicine, Tianjin 300192, China*

*^3^ National Clinical Research Center for Chinese Medicine Acupuncture and Moxibustion, Tianjin 300192, China*

*^4^ Tianjin University of Traditional Chinese Medicine, Tianjin 300193,* *China*

**^†^**YuJie Lu and Jie Ji contributed equally to this work.

***Corresponding author:** Min Jiang, Gang Bai

**Tel / Fax:** +86-022-23506930 (M. Jiang, G. Bai)

**E-mail address:** minjiang@nankai.edu.cn (M. Jiang) & gangbai@nankai.edu.cn (G. Bai)

**1. Supplementary methods**

***1.1 Inclusion criteria and treatment of patients***

Hospitalized patients with atherosclerosis, aged 50-70 years, who had a clear history of myocardial infarction, who were either diagnosed with coronary stenosis greater than 50% or were stable for more than a month after the treatment for acute coronary syndrome, were selected. These patients had not taken any TCM or Chinese patent drugs in the 2 weeks before enrollment. All patients provided signed informed consent. Subsequent clinical trials were conducted at the First Affiliated Hospital of Tianjin University of Traditional Chinese Medicine (Tianjin, China). Ethical approval for human and animal experiments was granted by the ethics committee of the First Affiliated Hospital of Tianjin University of Traditional Chinese Medicine. (Ethical approval number: TYLL2017[K]No.015).

A single-center, randomized, controlled, evaluator-blind method was used to design the clinical trial. All patients, who were randomly divided into two groups, received routine basic treatment, including anticoagulation, antiplatelet therapy, blood pressure control, blood glucose control, and other symptomatic treatments. Patients in the SX group were treated with SX (batch number 610029) based on routine treatment, and the dosage was 6 pills at a time, thrice a day, administered sublingually. Patients in the At-Ca group were treated with atorvastatin calcium tablets (Pfizer Pharmaceuticals, batch number R03220105, New York, NY, USA) based on the routine treatment, and the dosage was one 10 mg tablet to be orally taken once daily for two weeks. Infection markers, such as neutrophils (NEUT), lymphocytes (LYMPH), and the ratio of neutrophils to lymphocytes (NEUT/LYMPH), and other indicators, such as low-density lipoprotein (LDL), high-density lipoprotein (HDL), and total cholesterol (TC) were tested and evaluated in all patients using a biochemical analyzer (Hitachi 7600, Tokyo, Japan). Subsequently, 10 patients were randomly selected from each group, and the cardiac function before and after treatment was measured using color Doppler echocardiography (GE LOGIQ E9, Boston, Massachusetts, USA). Furthermore, after 15 min of administration, the indices after treatment were tested.

***1.2 Cell culture***

Both human vascular smooth muscle cells (T/G HA-VSMCs) and human embryonic kidney cells (293T) were purchased from American Type Culture Collection (Manassas, VA, USA) and were cultured in DMEM supplemented with 10% fetal bovine serum, 100 U/mL penicillin G, and 100 mg/mL streptomycin at 37 °C and 5% CO_2_ in a thermostatic incubator.

***1.3 iTRAQ-based differential proteomic analysis***

SX and pharmaceutical preparations corresponding to active pharmaceutical ingredient (API) extract were kindly provided by the No. 6 TCM factory of Tianjin Zhongxin Pharmaceutical Group Co., Ltd. (Tianjin, China). T/G HA-VSMCs were cultured in 10-cm culture plates. When the cells reached approximately 80% confluence, they were lysed in RIPA lysis buffer (containing protease inhibitor cocktail). The cell lysates were collected and divided into two parts, one of which was supplemented with SX API extract at a final concentration of 1 mg/mL and the other with an equal volume of vehicle. The samples were incubated at 4 °C for 12 h. After incubation, the two groups of samples were heated at 56 °C for 4 min and then cooled at 25 °C for 3 min. Subsequently, the supernatants were collected for the iTRAQ-based proteomic analysis. The differentially expressed proteins between the two groups were identified and quantified by Q-Exactive HF X (Thermo Fisher Scientific, San Jose, CA, USA) after digestion with trypsin and labeling with iTRAQ reagent.

***1.******4*** ***CaMKII-mediated dual-luciferase reporter assays for calcium antagonists***

The UPLC fractions of the SX extract were collected every 30 s for dual-luciferase assay. The 293T cells were co-transfected with CaMKII2G plasmid (CH826586, WeiZhen biosciences, Shandong, China), Ca^2+^ luciferase reporter plasmid PGL 4.30, and Renilla luciferase plasmid (50:25:1, m/m/m) for 22 h, according to our previously reported method^18^. The cells were simultaneously treated with ionomycin (Ion, 1 mM) and phorbol 12-myristate 13-acetate (PMA, 1 mg/mL) for 6 h. The cells were then tested using a dual-luciferase reporter assay, according to the manufacturer's instructions (E1960, Promega, Madison, WI, USA). The evaluation of CaMKII-mediated CCBs of screened monomer compounds was carried out using the same method. KN-93 (Aladdin, Shanghai, China), and its chemical name is (2-[N-(2-hydroxyethyl)]-N-(4-methoxybenzenesulfonyl)] amino-N-(4-chlorocinnamyl)-N methyl benzylamine), a recognized CaMKII inhibitor, served as a positive control in the experiment.

***1.5 In-gel fluorescence imaging***

An alkynyl-modified Lig probe was synthesized and evaluated. To determine the pharmacophore of butyl phthalides, the cells were treated with Lig, Sen A, and Sen I (10 μM, Macklin, Shanghai, China), and all were simultaneously administered with Lig probe (1 μM). After 24 h, the cells were collected and lysed. The lysates were incubated with the click reaction solution (10 μM BDP TMR azide (42430, Lumiprobe, Hunt Valley, MD, USA), 1 μM Cu_2_SO_4_, 1 μM TCEP, and 0.1 μM TBTA) for 1 h at 37 °C to obtain Lig-modified fluorescent product. After washing with methanol to remove the unbound fluorophore tracer, the protein precipitates were dissolved in 0.2% sodium dodecyl sulfate (SDS) and separated by SDS-PAGE. For in-gel fluorescence imaging, the gels were scanned on a Tanon-5200 Multi Gel Imaging System (Tanon 8 Science & Technology, Shanghai, China) for the BDP-Lig probe-labeled protein. The gels were transferred to PVDF membranes for western blotting, and CaMKII was used as an internal reference to analyze the relative fluorescence intensity.

***1.6 Fluorescence co-localization of CaMKII and Lig probe***

The VSMCs were treated with Lig probe (1 μM) with or without Lig (10 μM) for 24 h. Then, the cells were fixed with 4% paraformaldehyde. After blocking with 5% goat serum for 1 h, the cells were incubated with the CaMKII antibody (1:200, bs2052, Bioworld, Bloomington, MN, USA) at 4 °C overnight and then with Alexa Fluor® 594-conjugated goat anti-rabbit H&L (1:1000, ab150080, Abcam, Cambridge, UK) for 1 h at 25 °C. Lastly, the click reaction was carried out with BDP TMR azide (10 μM) to indicate the location of Lig, according to the method described above. Fluorescence imaging was performed using a laser scanning confocal microscope, Leica SP8 (Carl Zeiss, Oberkochen, Germany). For imaging the molecular targets in tissue, the mice were treated with Lig (i.p. 40 mg/kg/day) or Lig probe (i.p. 40 mg/kg/day) for 3 days, and the thoracic aortic sections were prepared and incubated with CaMKII antibody and BDP TMR azide, as described above.

***1.7 Cellular thermal shift assay (CETSA)***

For CETSAs, the cell lysate was incubated with the SX extract at a final concentration of 1 mg/mL, or the same volume of vehicle at 4 °C for 12 h, as described above. After incubation, the two groups of samples were divided into eight equal parts. The eight samples in each group were then heated individually at different temperatures (40, 44, 48, 52, 56, 60, 64, and 68 °C) for 4 min, followed by cooling at 25 °C for 3 min. After centrifugation, the supernatants were collected for western blot analysis.

For the dose-dependent thermal shift assay of SX or the active compound, the obtained T/G HA-VSMC cell lysates were incubated with different concentrations of SX extract (0.005, 0.025, 0.05, 0.1, 0.25, 0.5, and 1 mg/mL), or three bioactive compounds, Lig, Sen A, and Sen I (0.3, 0.6, 1.25, 2.5, 5, 10, and 20 μM) at 4 °C for 12 h, respectively. Subsequently, all samples were heated at 53 °C for 4 min and then cooled at 25 °C for 3 min. The remaining experimental procedures were the same as described above.

***1.8 Quantitative analyses of*** ***SX extract***

An ACQUITY UPLC® CSH^TM^ C18 (1.7 μm, 2.1×100 mm) and a Waters ACQUITY UPLC System were used to separate and detect the components of SX extract. The injection volume was 2 μL, and the column temperature was maintained at 35°C. The mobile phase was a gradient elution system of A (H_2_O) and B (CH_3_CN, Merck, Darmstadt, Germany), and the elution was programmed as follows: 5-10% B for 0-2 min, 10-20% B for 2-4 min, 20-24% B for 4-6 min, 24-38% B for 6-20 min, 38-60% B for 20-23 min, 60-100% B for 23-26 min, and 100% for 26-27 min. The flow rate was 0.4 mL/min. The detection wavelength was 290 nm. Under the same conditions, the qualitative and quantitative analysis of Lig and Sen A in SX extract were carried out using chemical standards respectively.

***1.9 Expression and purification of CaMKIIγ***

Briefly, the CaMKIIγ gene (the catalytic domain of CaMKIIγ, residues 1-271), which contains 813 bases was cloned into the pET-32a (+) expression vector, and the 6×his tag was fused at the C-terminus. The expression vector was transformed with E. coli BL21 (DE3) strain and induced with 0.2 mmol/L of isopropyl β-D-1-thiogalactoside (IPTG) at an optical density at 600 nm of 0.6. After 16 °C overnight expression, the cultured protein-expressing cells were collected in a buffer consisting of 50 mM Tris HCl (pH 7.8), 200 mM NaCl and 5% glycerol, and stored at -80 °C until use. When purifying the protein, the cells were disrupted by an ultrasonic apparatus, then centrifugated at 13,000 g for 15 mins at 4 °C. The supernatant was applied to a Ni-NTA column for affinity adsorption. Washing with a buffer containing 30 mM imidazole, 50 mM Tris HCl (pH 7.8), 200 mM NaCl and 5% glycerol, eluting with a buffer containing 400 mM imidazole, 50 mM Tris HCl (pH 7.8), 200 mM NaCl and 5% glycerol. Subsequently, thioredoxin was removed from the N-termini of the proteins with enterokinase. The proteins were applied to a Ni-NTA column for affinity adsorption once again to remove enterokinase. The purified CaMKIIγ protein was analyzed by SDS-PAGE and western blot.

***1.10 Identification of Lig-binding site on CaMKIIγ***

The recombinant wild-type human CaMKIIγ (residues 1-271), recombinant C116 mutant protein (C116G), and C148 mutant protein (C148G), in which cysteine was substituted with glycine, were expressed in the *E. coli* expression system.

The recombinant CaMKIIγ was incubated with E-Lig for 12 h at 4 °C, and the reactions were resolved by SDS-PAGE. Bands corresponding to CaMKIIγ were excised, and digested in gel with trypsin. Extracted peptides were auto-sampled directly and loaded onto C18 reversed-phase analytical column (2μm, 75μm×25cm, nanoViper, Thermo Fisher Scientific). The injection volume was 2 μL. The mobile phase was 0.1% formic acid in water (solvent A) and 0.1% formic acid in acetonitrile (solvent B) and the elution was programmed as follows: 3-8% B for 0-5 min, 8-18% B for 5-75 min, 18-28% B for 75-103 min, 28-90% B for 103-115 min, 90-90% B for 115-120 min, all at a constant flow rate of 300 nL/min on an EASY-nLC 1000 UPLC system (Thermo Fisher Scientific). The peptides were subjected to NSI source followed by tandem mass spectrometry (MS/MS) in Orbitrap Fusion TM (Thermo Fisher Scientific) coupled online to the UPLC. The electrospray voltage applied was 2.4 kV. Full scan MS spectra (from m/z 350 to 2000) were acquired in the Orbitrap analyzer with a resolution of 120,000. Peptides were then selected for MS/MS scans using HCD Collision Energy setting as 30%. A data-dependent procedure was performed using a top-speed approach (cycle time of 3.0 s) with 15.0 s dynamic exclusion. Automatic gain control (AGC) target was set to standard.

The Mass spectrometric data were analyzed with Proteome Discoverer 1.4 software (Thermo Fisher Scientific). Trypsin was specified as cleavage enzyme allowing up to 2 missing cleavages. The mass error of precursor ions was set to 10 ppm and the mass error of fragment ions was set to 0.02 Da. Peptide confidence was set at high.

***1.11 Cardiac function evaluation in mice***

Male C57BL/6J mice and male ApoE^−/−^ mice, 8 weeks of age, were purchased from Vital River Laboratory Animal Technology Co., Ltd. (Beijing, China) and acclimated to laboratory conditions for one week before the experiment began. They were fed a high-fat diet containing 21% fat and 1.5% cholesterol in basal mouse chow. Food and water were provided ad libitum. The mice were housed in an air-conditioned room at a constant temperature (23 ± 2 °C), humidity (45-50%), under diurnal illumination of 12 h light/dark (L/D) cycles. The ApoE^−/−^ mice were randomly assigned into six groups (*n*=6): model, positive, SX, and high-, medium-, and low-dose Lig groups that were treated with normal saline, At-Ca (3 mg/kg/d), SX (270 mg/kg/d), and 20, 10, and 5 mg/kg/d of Lig, respectively. After 12 weeks of intragastric administration, the cardiac function of the mice was assessed using a Vevo 2100 Imaging System (VisualSonics, Canada).

***1.12 Vasodilatory effect test***

Eight-week-old male Sprague-Dawley (SD) rats were purchased from Vital River Laboratory Animal Technology Co., Ltd. (Beijing, China). The thoracic aorta was carefully dissected from the rats, and the endothelial tissue was removed. Subsequently, the thoracic aorta was cut into approximately 3.0 mm arterial rings and suspended in a 37 °C thermostatic water bath (Radnoti Tissue-Organ Bath System, Covina, CA, USA) containing 10 mL of Kreb-Henseleit solution while being continuously injected with 95% oxygen and 5% carbon dioxide. To measure the vasodilatory activity of Lig, a 60 mmol/L potassium chloride (KCl) solution was employed for inducing stable vasoconstriction. Vascular relaxation function was detected using 1 μmol/L of KN-93 and different concentrations of Lig (10, 1, and 0.1 μmol/L). To further verify the relationship between vasodilator activity and CaMKII, the dilated thoracic aortas, after administration for 20 min, were immediately ground and lysed in RIPA lysis buffer containing 1% protease inhibitor cocktail and 1% protein phosphatase inhibitor mixture. The supernatants were collected for CaMKII and MLC phosphorylation analyses by western blot.

***1.13 Chemicals and reagents***

Suxiao Jiuxin pills (SX) with batch number 610029 and SX extract were kindly provided by the No. 6 TCM factory of Tianjin Zhongxin Pharmaceutical Group Co., Ltd. (Tianjin, China). Atorvastatin calcium tablets (At-Ca) were purchased from Pfizer Pharmaceuticals (batch number R03220105, New York, NY, USA). Ligustilide (Lig), senkyunolide A (Sen A) and senkyunolide I (Sen I) were obtained from Macklin (Shanghai, China), Selleck Chemicals (Houston, TX, USA), and Solarbio (Beijing, China), respectively. BDP TMR azide (42430), 6, 7-epoxyligustilide (E-Lig), and KN-93 were purchased from Lumiprobe (Hunt Valley, Maryland, USA), Pufei De Biotech (Chengdu, Sichuan, China), and Aladdin (Shanghai, China), respectively. The purity of all the above chemical reagents was ≥ 98%, as determined by HPLC. The CaMKII β/γ antibody (bs2052) was purchased from Bioworld Technology (Bloomington, MN, USA). p-CaMKII (T287) antibody (ab182647), anti-GAPDH (ab1802), Alexa Fluor®594-conjugated goat anti-rabbit H&L (ab150080), and Alexa Fluor® 488-conjugated goat anti-mouse IgG H&L (ab150113) were obtained from Abcam (Cambridge, UK). Anti-mouse p-CaMKII (sc-32289) was obtained from Santa Cruz Biotechnology (Dallas, TX, USA). Myosin light chain (MLC, 3672S) and p-MLC (3671S) antibodies were purchased from Cell Signaling Technology (Beverly, MA, USA). All reagents for cell culture were purchased from Gibco/Invitrogen (Carlsbad, CA, USA).

***1.14 Molecular docking***

The 3D structure of the CaMKIIγ protein was obtained from the Protein Data Bank (http://www1.rcsb.org/structure/2V7O). The protein preparation wizard module in the Schrödinger software (Schrodinger, Inc.) was used to optimize CaMKIIγ structure. The LigPrep module in the Schrödinger software was used to optimize the ligand of E-Lig. The covalent docking module in the Schrödinger software was used to covalently dock the optimized compound into the target protein active site. The reaction type was set to epoxide opening, and the other parameters remained at their default values.

***1.15*** ***Immunohistochemical analysis***

For immunohistochemical experiments, 5-mm thick paraffin sections of the mouse thoracic aorta were prepared. After dewaxing, antigen repair, and removal of endogenous enzymes, the sections were blocked with 5% goat serum for 1 h at 25 °C. Subsequently, the sections were incubated with mouse p-CaMKII monoclonal antibody (1:200) and rabbit CaMKII polyclonal antibody (1:200) at 4 °C for 12 h. Finally, the sections were labeled with Alexa Fluor® 488-conjugated goat anti-mouse IgG H&L (1:1000) and Alexa Fluor® 594-conjugated goat anti-rabbit IgG H&L (1:1000). The images were acquired using a laser scanning confocal microscope Leica SP8 (Carl Zeiss, Oberkochen, Germany).

***1.16*** ***Western blot***

The proteins were separated by SDS-PAGE, transferred to PVDF membranes, blocked with 5% skim milk, and incubated with primary antibodies (CaMKII, p-CaMKII, MLC, p-MLC, and GAPDH) for 12 h at 4 °C, followed by incubation with horseradish peroxidase-labeled secondary antibodies at 25 °C for 1 h. The immune complexes were visualized using a chemiluminescence imaging system (Tanon-5200, Tanon, Shanghai, China).

***1.17 Statistical analysis***

Data analysis was performed using GraphPad Prism 6.0 (GraphPad Software Inc., San Diego, CA, USA). All experimental results are presented as the mean ± SD and were obtained from at least three independent experiments. Student’s *t*-test or one-way analysis of variance (ANOVA) was used to analyze the significance of the intergroup differences. *P*<0.05 was considered statistically significant.

**2. Supplementary figures**

**2.1 Table S1**

**Table S1 Baseline characteristics of 100 patients included in the analyses.**

| Variable and category | Condition | | Total (n=100) |
| --- | --- | --- | --- |
|  | SX (n=50) | At-Ca (n=50) |  |
| Age, years |  |  |  |
| 50-60 | 18 (36%) | 17 (34%) | 35 (35%) |
| 61-70 | 32 (64%) | 33 (63%) | 65 (65%) |
| Sex |  |  |  |
| Male | 32 (64%) | 37 (74%) | 69 (69%) |
| Female | 18 (36%) | 13 (26%) | 31 (31%) |
| Education category |  |  |  |
| Never attended school | 3 (6%) | 0 (0%) | 3 (3%) |
| Primary school | 4 (8%) | 6 (12%) | 10 (10%) |
| Middle school | 40 (80%) | 39 (78%) | 79 (79%) |
| University or above | 3 (6%) | 5 (10%) | 8 (8%) |
| Job |  |  |  |
| Yes | 12 (24%) | 5 (10%) | 17 (17%) |
| No | 38 (76%) | 45 (90%) | 83 (83%) |
| Relationship status |  |  |  |
| Single | 0 (0%) | 2 (4%) | 2 (2%) |
| Married | 33 (66%) | 37 (74%) | 70 (70%) |
| Divorced | 12 (24%) | 6 (12%) | 18 (18%) |
| Other | 4 (8%) | 5 (10%) | 9 (9%) |
| Family history |  |  |  |
| Yes | 25 (50%) | 21 (42%) | 46 (46%) |
| No | 25 (50%) | 29 (58%) | 54 (54%) |
| Drinking |  |  |  |
| Never | 31 (62%) | 29 (58%) | 60 (60%) |
| Occasionally | 6 (12%) | 7 (14%) | 13 (13%) |
| Often | 13 (26%) | 14 (28%) | 27 (27%) |
| Smoking |  |  |  |
| Never | 27 (54%) | 26 (52%) | 53 (53%) |
| Occasionally | 5 (10%) | 2 (4%) | 7 (7%) |
| Often | 18 (36%) | 22 (44%) | 40 (40%) |
| Diet |  |  |  |
| Unbiased | 41 (82%) | 43 (86%) | 84 (84%) |
| Partiality | 9 (18%) | 7 (14%) | 16 (16%) |
| Sleep |  |  |  |
| Good | 35 (70%) | 30 (60%) | 65 (65%) |
| Poor | 11 (22%) | 17 (34%) | 28 (28%) |
| Insomnia | 4 (8%) | 3 (6%) | 7 (7%) |
| Stress of spiritual life |  |  |  |
| Large | 7 (14%) | 4 (8%) | 11 (11%) |
| Medium | 12 (24%) | 16 (32%) | 28 (28%) |
| Small | 31 (62%) | 30 (60%) | 61 (61%) |

**2.2 Figure S1**


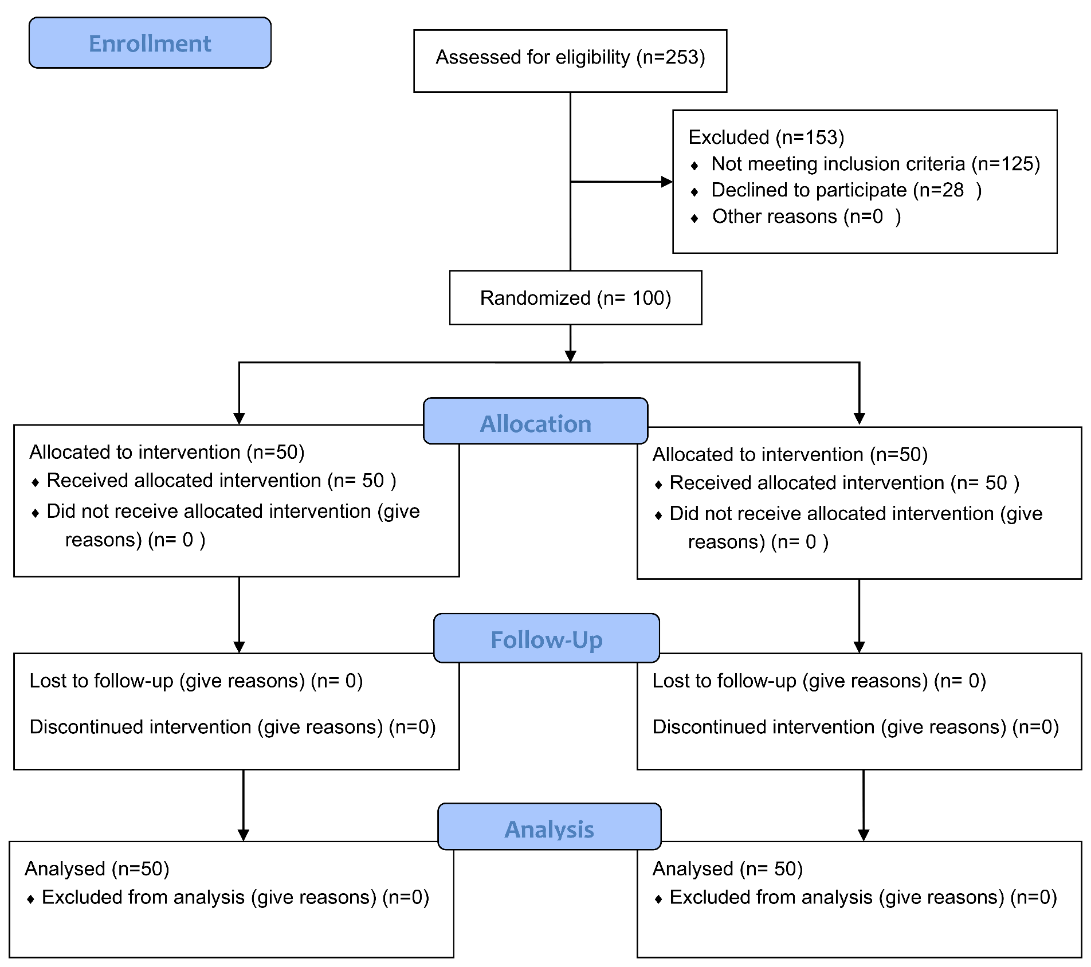


Figure S1. Enrollment of Participants and Study Flow

**2.3 Table S2**

Table S2 Baseline characteristics of 10 patients in each group.

| Variable and category | Condition | | Total (n=20) |
| --- | --- | --- | --- |
|  | SX (n=10) | At-Ca (n=10) |  |
| Age, years |  |  |  |
| 50-60 | 2 (20%) | 6 (60%) | 8 (40%) |
| 61-70 | 8 (80%) | 4 (40%) | 12 (60%) |
| Sex |  |  |  |
| Male | 8 (80%) | 7 (70%) | 69 (69%) |
| Female | 2 (20%) | 3 (30%) | 31 (31%) |
| Education category |  |  |  |
| Never attended school | 1 (10%) | 0 (0%) | 1 (5%) |
| Primary school | 1 (10%) | 0 (0%) | 1 (5%) |
| Middle school | 8 (80%) | 10 (100%) | 18 (90%) |
| University or above | 0 (0%) | 0 (0%) | 0 (0%) |
| Job |  |  |  |
| Yes | 4 (40%) | 1 (10%) | 5 (25%) |
| No | 6 (60%) | 9 (90%) | 15 (75%) |
| Relationship status |  |  |  |
| Single | 0 (0%) | 0 (0%) | 0 (0%) |
| Married | 5 (50%) | 5 (50%) | 10 (50%) |
| Divorced | 0 (0%) | 0 (0%) | 0 (0%) |
| Other | 5 (50%) | 5 (50%) | 10 (50%) |
| Family history |  |  |  |
| Yes | 6 (60%) | 3 (30%) | 9 (45%) |
| No | 4 (40%) | 7 (70%) | 11 (55%) |
| Drinking |  |  |  |
| Never | 4 (40%) | 6 (60%) | 10 (50%) |
| Occasionally | 1 (10%) | 2 (20%) | 3 (15%) |
| Often | 5 (50%) | 2 (20%) | 7 (35%) |
| Smoking |  |  |  |
| Never | 4 (40%) | 3 (30%) | 7 (35%) |
| Occasionally | 0 (0%) | 1 (10%) | 1 (5%) |
| Often | 6 (60%) | 6 (60%) | 12 (60%) |
| Diet |  |  |  |
| Unbiased | 9 (90%) | 8 (80%) | 17 (85%) |
| Partiality | 1 (10%) | 2 (20%) | 3 (15%) |
| Sleep |  |  |  |
| Good | 10 (100%) | 6 (60%) | 16 (80%) |
| Poor | 0 (0%) | 4 (40%) | 4 (20%) |
| Insomnia | 0 (0%) | 0 (0%) | 0 (0%) |
| Stress of spiritual life |  |  |  |
| Large | 0 (0%) | 0 (0%) | 0 (0%) |
| Medium | 5 (50%) | 5 (50%) | 10 (50%) |
| Small | 5 (50%) | 5 (50%) | 10 (50%) |

**2.4 Figure S2**

**
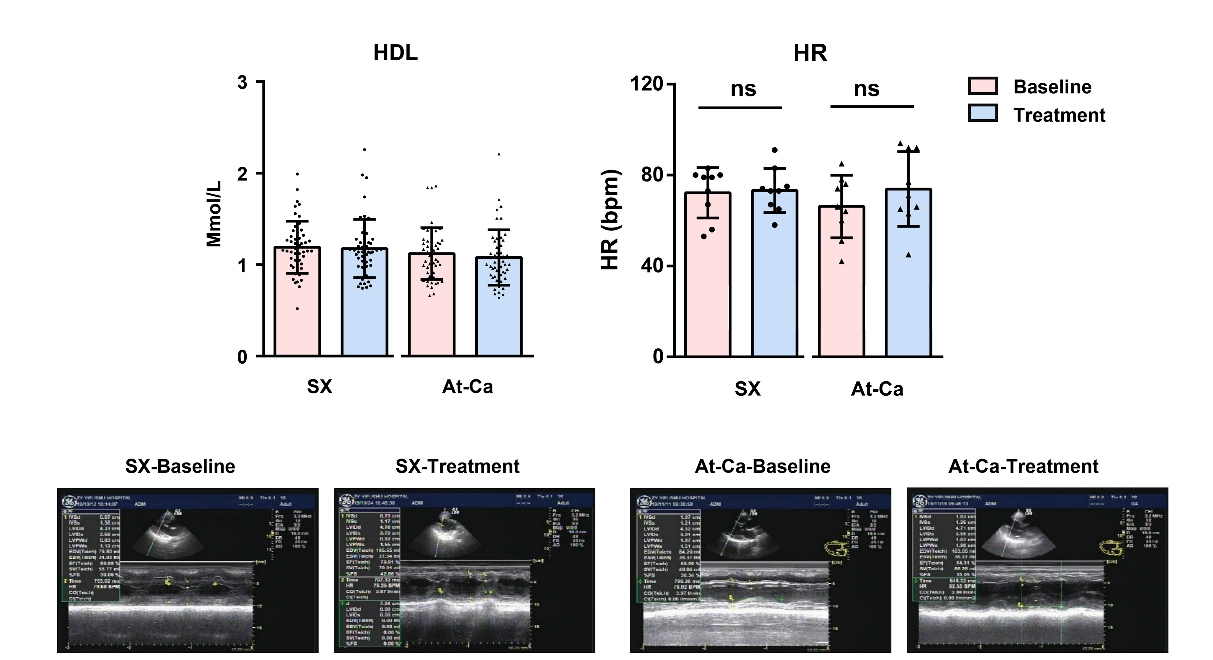
**

**Figure S2.** Patients treated with SX (6 pills a time, 40 mg a pill, 3 times a day, sublingually) or atorvastatin calcium tablets (At-Ca, 10 mg tablet a time, once a day, orally) for 2 weeks underwent high-density lipoprotein (HDL) test (*n* = 50), and heart rate (HR) and representative M-mode images of patients were measured using color Doppler echocardiography (*n* = 10), before and after treatment.

**2.5 Figure S3**


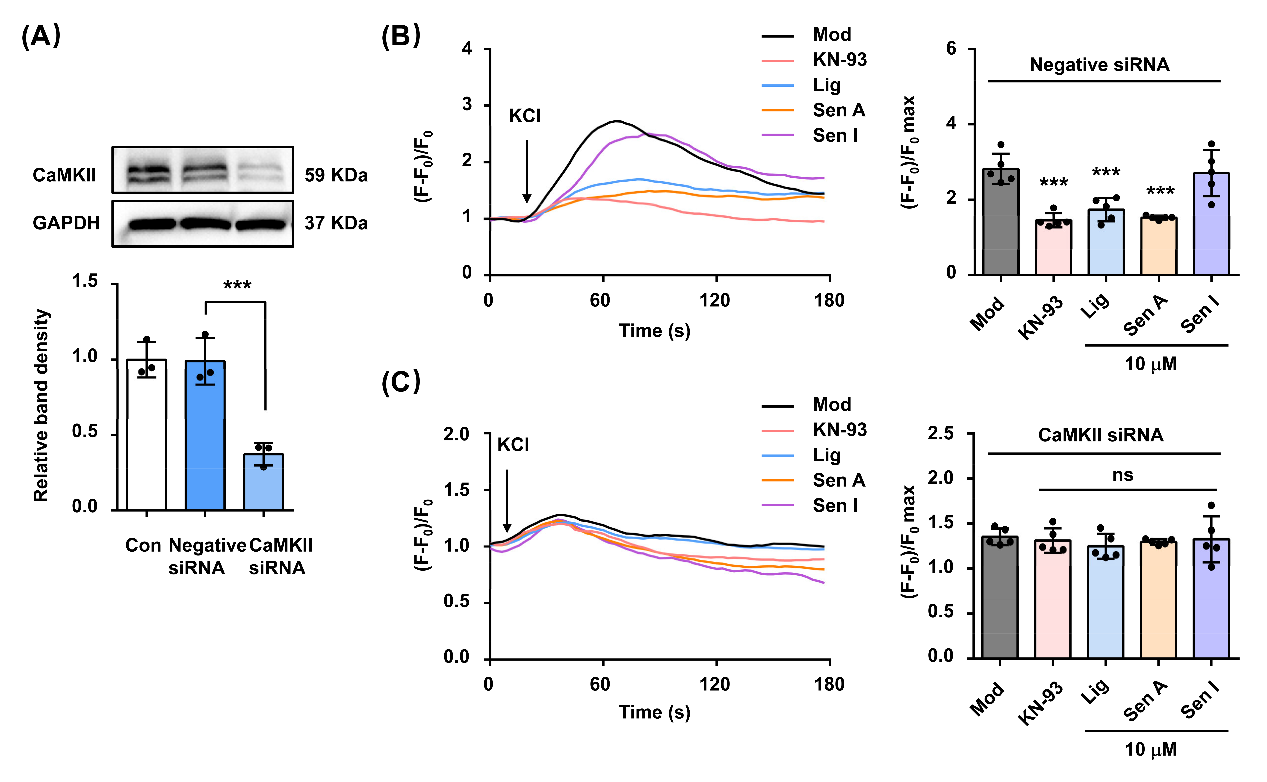


**Figure S3.** siRNA interference assay combined with live-cell imaging analysis reveal that the activity of Lig is mediated by CaMKII. **(A)** The prevention of CaMKII protein expression using a specific CaMKII siRNA. Western blot analysis was performed after transfection of VSMC cells with CaMKII siRNA for 48 h. Data are expressed as mean ± SD, n=3; *^***^P*<0.001 *vs* negative siRNA. Blocking effect of compounds on calcium channels by live‐cell imaging analysis in VSMC cells, which was induced by KCl in the absence **(B)** or presence **(C)** of CaMKII siRNA-transfection. The left panel represents the time-dependent variation trend of relative fluorescence intensity (RFI) in each group after stimulation with KCl; the right panel is a bar graph showing the maximum RFI in different groups. Data are expressed as mean ± SD, n=5; *^***^P*<0.001 *vs* Mod group; ns, no significant.

**2.6 Figure S4**


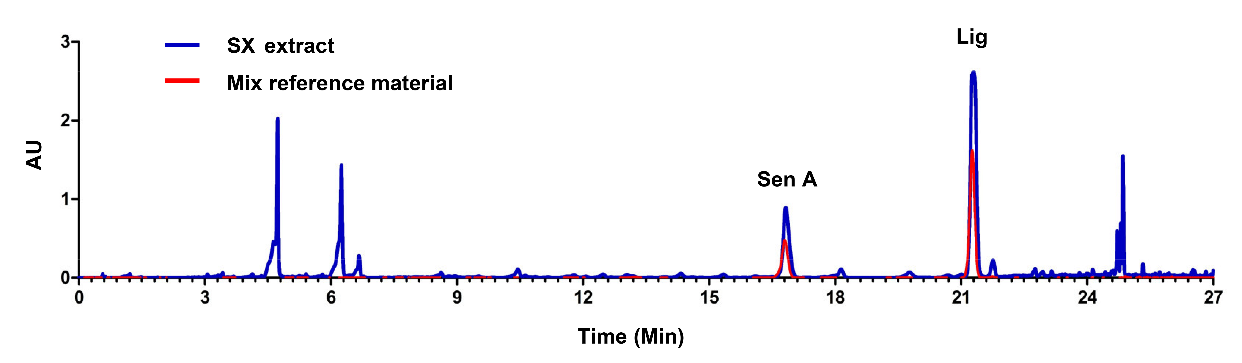


**Figure S4.** Lig and SenA in SX extract was analyzed by UPLC.

**2.7 Table S3**

**Table S3** The bioactive components in SX extract were quantitatively analyzed.

| No. | RT/min | Components | Content (mg/mg) |
| --- | --- | --- | --- |
| 1 | 16.82 | Sen A | 0.0355 |
| 2 | 21.25 | Lig | 0.0465 |

**2.8 Figure S**5

**Figure S5.** The detailed synthetic route of Lig-PAL. The synthesis of Lig-PAL was delegated to Wuxi App Tec (Tianjin, China).

**2.9 Figure S6**


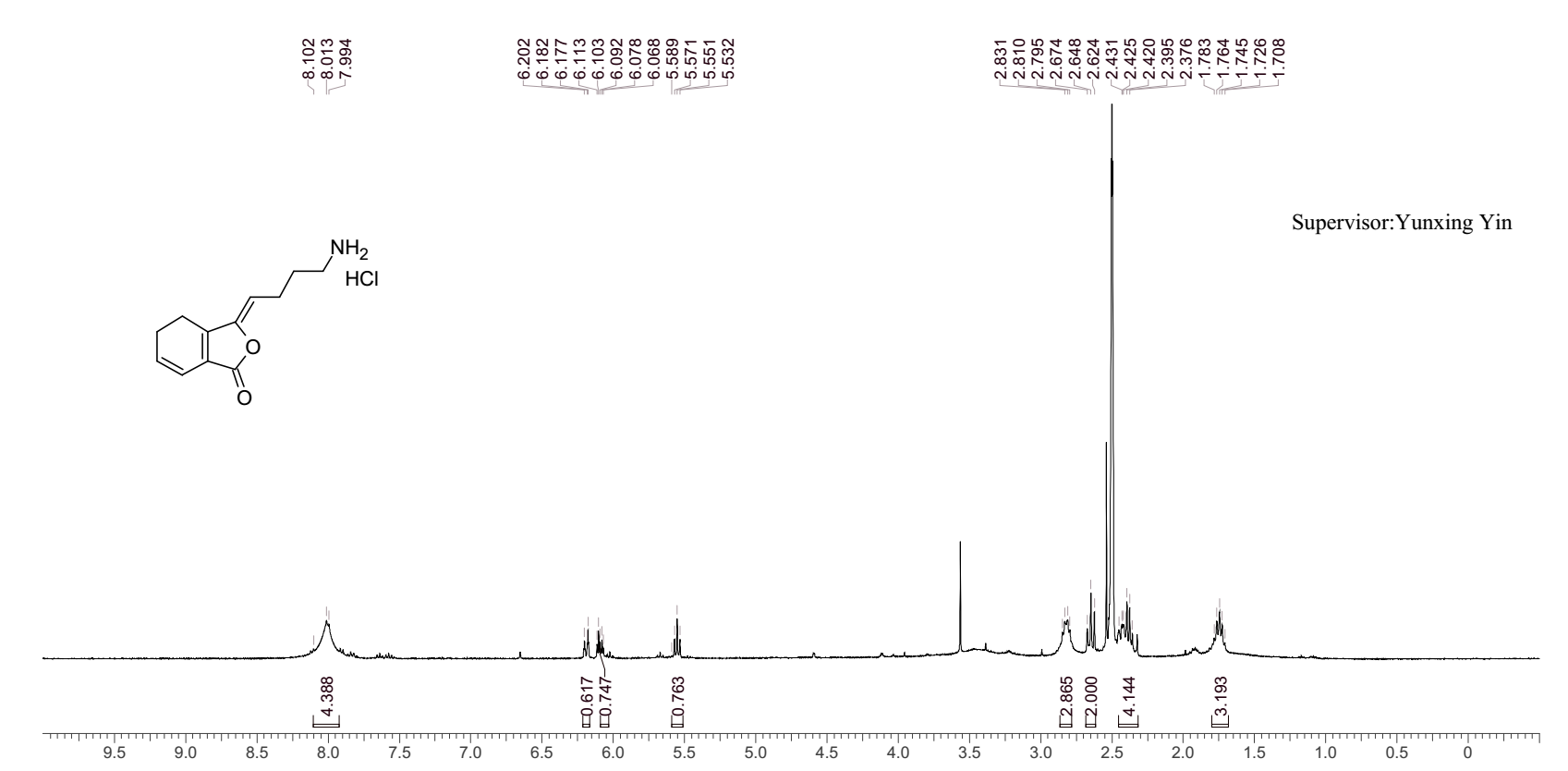


**Figure S6.** ^1^H NMR spectra for Lig-PAL**.** ^1^H NMR (400 MHz, DMSO-d_6_) *δ* 8.01 (s, 4H), 6.18-6.20 (m, 1H), 6.07-6.11 (m, 1H), 5.55 (t, J = 16 Hz, 1H), 2.80-2.83 (m, 3H), 2.65 (t, J = 20 Hz, 2H), 2.38-2.43 (m, 4H), 1.71-1.78 (m, 3H).

**2.10 Figure S7**


**Figure S7.** The detailed synthetic route of Lig probe.

**2.11 Figure S8**


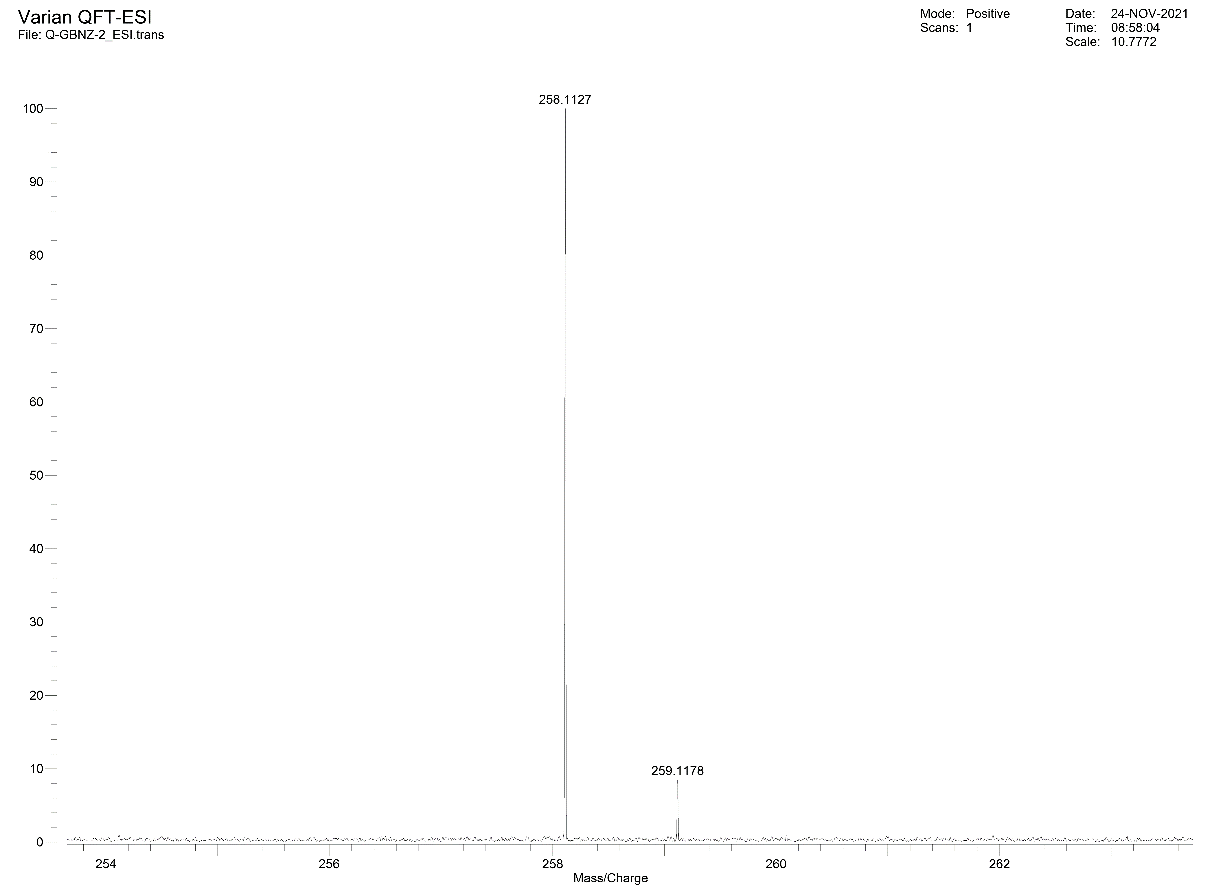


**Figure S8.** The high-resolution mass spectrometry (HRMS) of Lig probe.

**2.12 Figure S9**


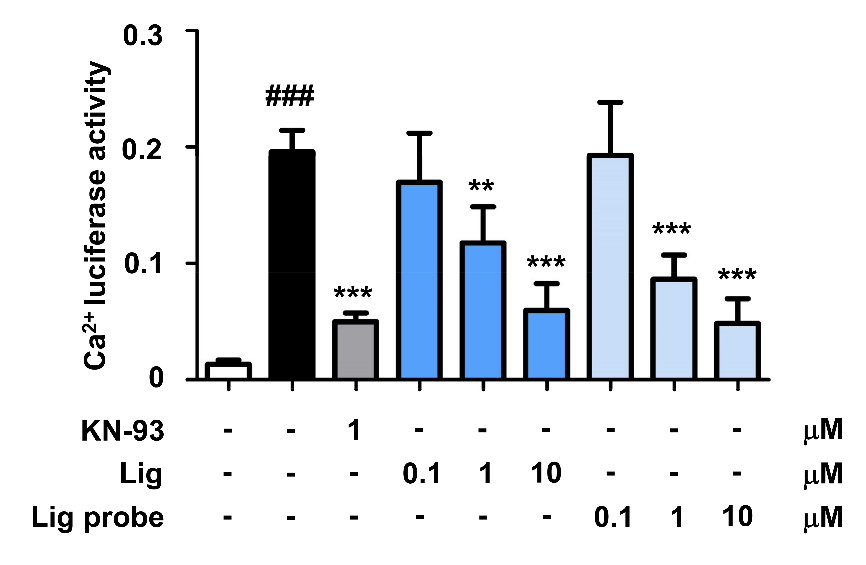


**Figure S9.** The effects of Lig and Lig probe on CaMKII-mediated Ca^2+^ antagonism. Data are expressed as mean ± SD, *n=4*; ^###^*P*<0.001 *vs*. Control; ^**^*P*<0.01 and ^***^*P*<0.001 *vs.* model group.

**2.13 Figure S10**


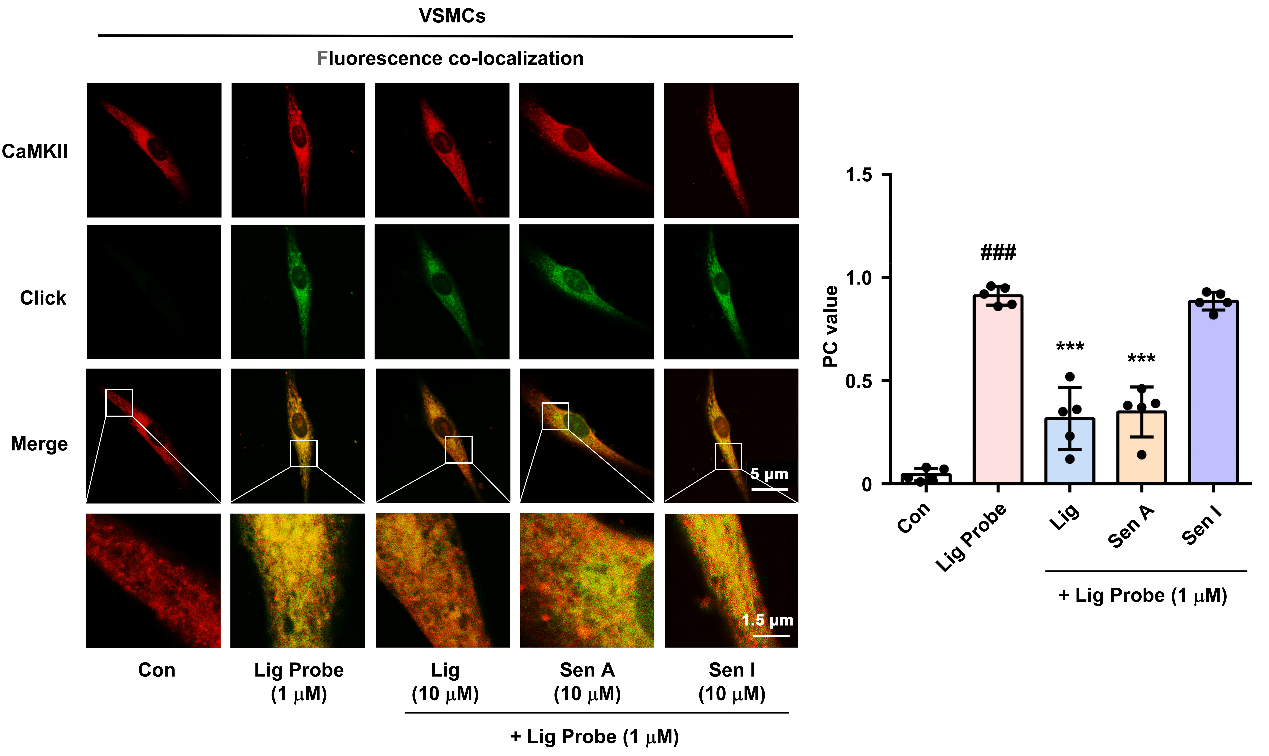


**Figure S10.** Co-localization of CaMKII (pseudo red) and Lig probe (pseudo green) in VSMCs, and Pearson coefficient (PC) analysis of the merged images.

**2.14 Figure S11**


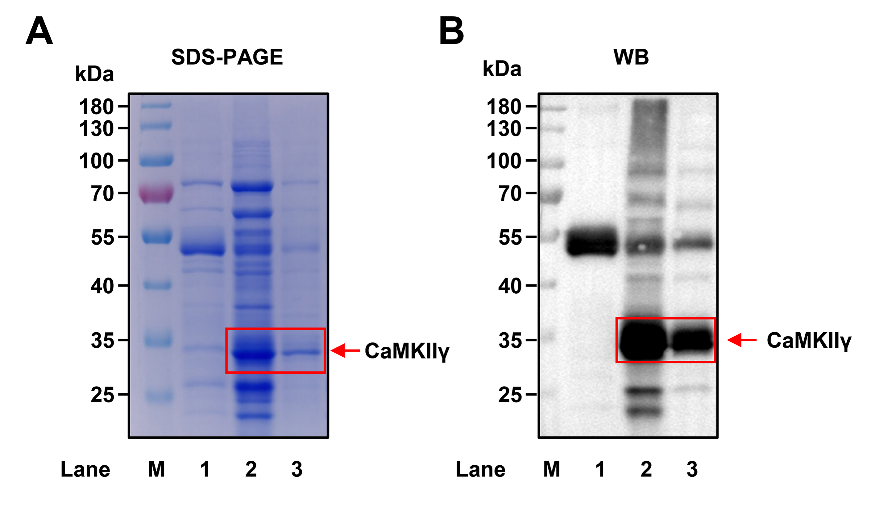


**Figure S11.** The expression and purification of CaMKIIγ protein (residues 1-271). (A) SDS-PAGE analysis of CaMKIIγ protein. M represents protein maker, Lane 1 was purified CaMKIIγ protein with thioredoxin, lane 2 was CaMKIIγ protein digested by enterokinase, lane 3 was CaMKIIγ protein purified by secondary affinity adsorption. (B) Detection of the CaMKIIγ protein by western blot analysis.

**2.15 Figure S12**

**
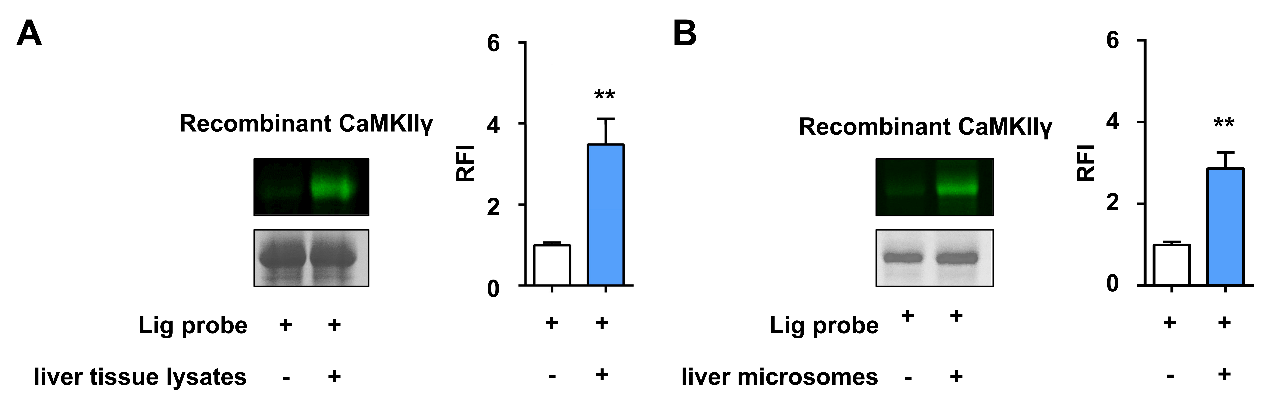
**

**Figure S12.** Lig binds to CaMKIIγ after metabolism in liver tissue and liver microsomes. The specific fluorescence of CaMKII in-gel was attenuated when there is no addition of liver tissue lysates and liver microsomes. Data are expressed as mean ± SD, n=3; **P<0.01 vs control group.

**2.16 Figure S13**


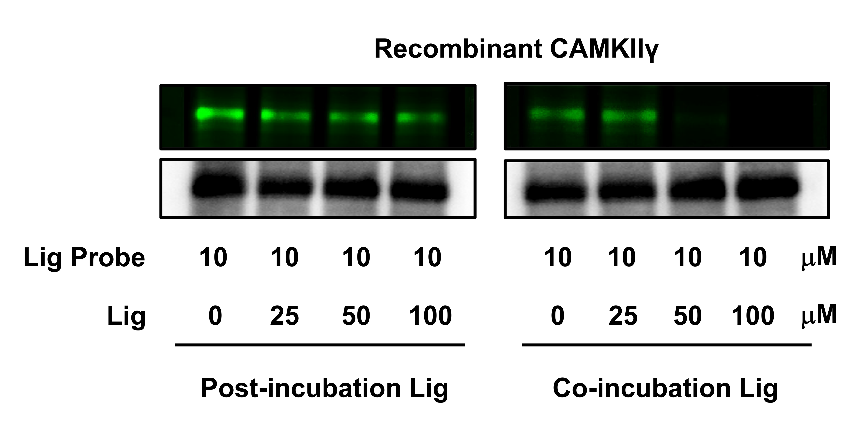


**Figure S13.** In-gel imaging for irreversible binding assay of Lig to CaMKIIγ. In the post-incubation group, the recombinant CaMKIIγ was pre-incubated with 10 μM Lig probe at 4 °C for 12 h, and was further incubated with 0-100 μM Lig for 12 h for competitive binding. In the co-incubation group, the recombinant CaMKIIγ was synchronously incubated with10 μM Lig probe and0-100 μM Lig for the competitive binding. Both groups were added to the liver tissue lysates (1 mg/mL) as the enzymatic reaction solution.

**2.17 Figure S14**


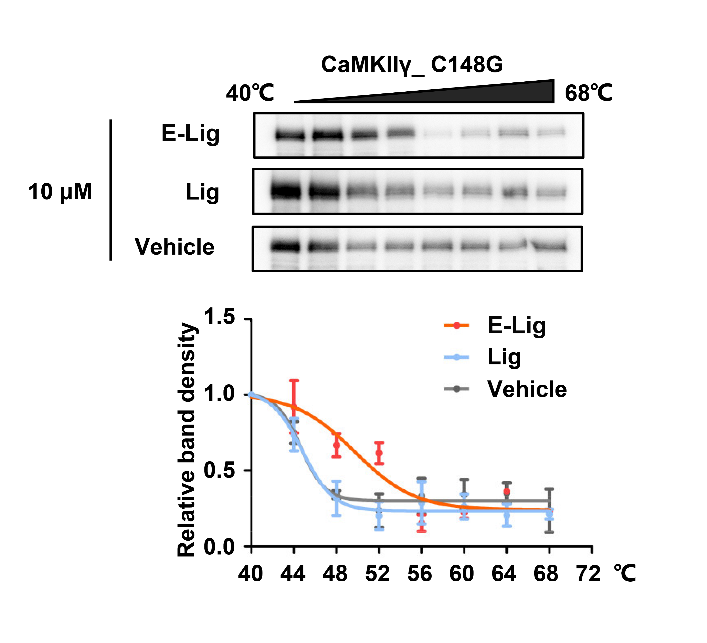


**Figure S14.** E-Lig metabolite increases the thermal stability of the C148G mutant CaMKIIγ, as measured by thermal shift assay (*n*=3).

**2.18 Figure S15**


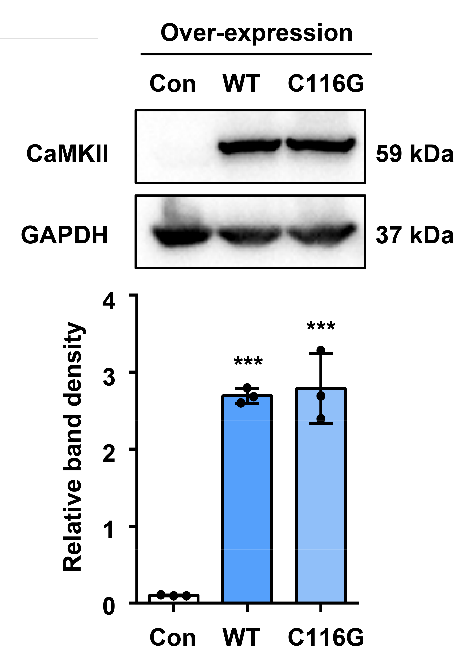


**Figure R15.** Transfection of WT and C116G CaMKII plasmid into HEK293 cells induces high-expression of WT and C116G protein. Data are expressed as mean ± SD, n=3; *^***^P*<0.001 *vs* negative plasmid.

**2.19 Figure S16**

**
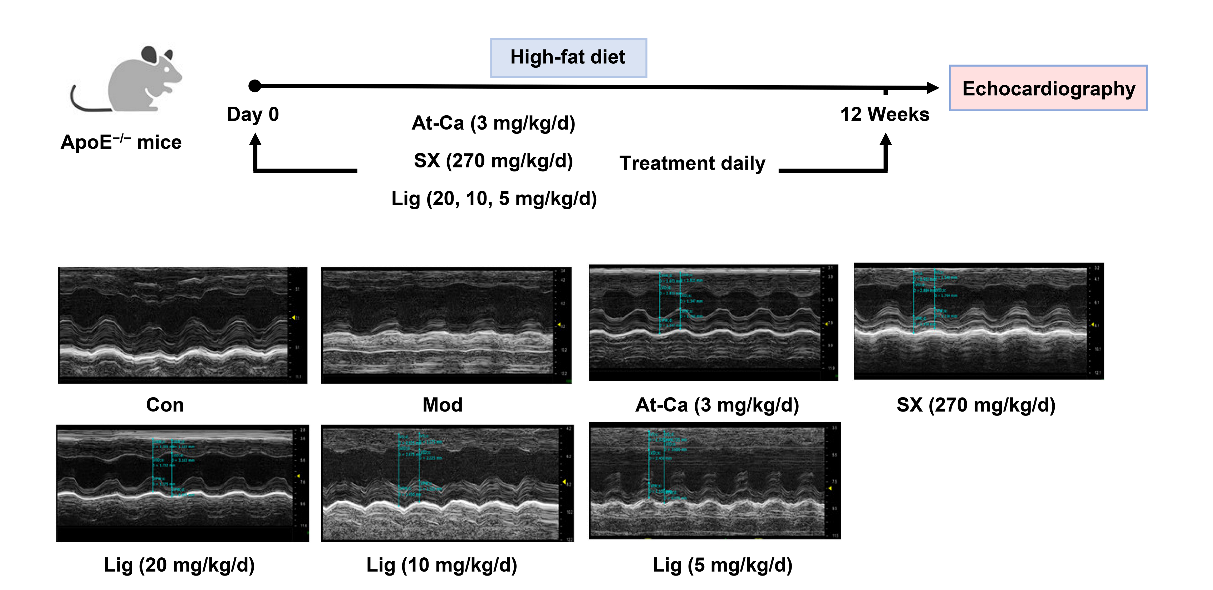
**

**Figure S16.** Experimental procedure and representative M-mode images were captured in wild-type control C57BL/6J mice treated with normal saline and ApoE^−/−^ mice treated with normal saline, At-Ca, SX and three doses of Lig, respectively.

**2.14 Figure S17**

**
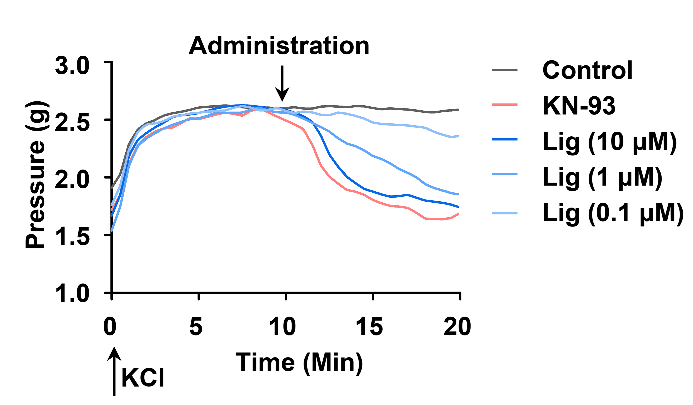
**

**Figure S17.** Time-dependent monitoring of vascular relaxation regulated by Lig and KN-93 in an isolated thoracic aorta of rats.
